# Vitamin B1 Intake in Multiple Sclerosis Patients and its Impact on Depression Presence: A Pilot Study

**DOI:** 10.3390/nu12092655

**Published:** 2020-08-31

**Authors:** Jose Enrique de la Rubia Ortí, María Cuerda-Ballester, Eraci Drehmer, Sandra Carrera-Juliá, María Motos-Muñoz, Cristina Cunha-Pérez, María Benlloch, María Mar López-Rodríguez

**Affiliations:** 1 Department of Nursing, Catholic University of Valencia San Vicente Martir, 46001 Valencia, Spain; maria.motos@ucv.es (M.M.-M.); cristina.cunha@ucv.es (C.C.-P.); maria.benlloch@ucv.es (M.B.); 2 Doctoral Degree School, Catholic University of Valencia San Vicente Martir, 46001 Valencia, Spain; m.cuerda@mail.ucv.es; 3 Department of Physical Activity and Sports Sciences, Catholic University San Vicente Martir, 46900 Valencia, Spain; eraci.drehmer@ucv.es; 4 Department of Nutrition and Dietetics, Catholic University of Valencia San Vicente Mártir, 46001 Valencia, Spain; sandra.carrera@ucv.es; 5 Department of Nursing, Physiotherapy and Medicine, University of Almería, 04120 Almería, Spain

**Keywords:** depression, multiple sclerosis, vitamin B1

## Abstract

Vitamin B1, or thiamine, is one of the most relevant vitamins in obtaining energy for the nervous system. Thiamine deficiency or lack of activity causes neurological manifestations, especially symptoms of depression, intrinsic to multiple sclerosis (MS) and related to its pathogenesis. On this basis, the aim of this study was to determine the possible relationship between the nutritional habits of patients with MS and the presence of depression. Therefore, a cross-sectional and observational descriptive study was conducted. An analysis of dietary habits and vitamin B1 consumption in a Spanish population of 51 MS patients was performed by recording the frequency of food consumption. Results showed a vitamin B1 intake within the established range, mainly provided by the consumption of ultra-processed products such as cold meats or pastries, and a total carbohydrate consumption lower than recommended, which stands out for its high content of simple carbohydrates deriving from processed foods such as dairy desserts, juice, snacks, pastries, chocolate bars, soft drinks and fermented alcohol. In addition, a significant negative correlation between depression and the intake of thiamine and total carbohydrates was observed. These findings could explain the influence of MS patients’ eating habits, and consequently vitamin B1 activity, on depression levels.

## 1. Introduction

Multiple sclerosis (MS) is a degenerative autoimmune disease of the central nervous system (CNS) characterized by inflammatory demyelination and axonal loss [1], being affected by mitochondrial function [2]. It represents the main cause of disability in young adults, with a higher prevalence in women (an approximate ratio of 3:1) [3]. MS mainly appears after the age of 30 [4] and usually firstly presents as relapsing-remitting, which over time becomes secondary-progressive in the majority of cases [5]. Regarding signs and symptoms, disability and fatigue are characteristic of the disease [6] and are usually accompanied by emotional disturbances [7]. Concerning these emotional problems, 50% of patients with MS have depression (2–3 times higher compared to the general population) [8]. Depression is directly related to functional disorders [9] and influences the pathogenesis of the disease [10].

Current drugs used to treat depression have many side effects [11]. Due to these side effects, an alternative treatment for depression could be a correct intake of foods associated with antidepressant effects, for example those provided by vitamin B1 or thiamine [12]. Emotional disturbances depend on the activity of the nervous system in which the correct functioning of B vitamins is important. Therefore, a balanced intake of these vitamins, especially B1, could be a possible strategy to prevent emotional disturbances related to neurodegenerative disease [13].

Vitamin B1 or thiamine is involved in an organism’s energy metabolism, processing carbohydrates into energy for the nervous system while also playing a fundamental role in growth, development and cell differentiation processes [14]. Thus, mitochondrial dysfunction is observed when thiamine-dependent processes are compromised. Such alteration at a mitochondrial level produces an increase in oxidative stress, inflammatory changes, a decrease of neurogenesis, and the interruption of the blood–brain barrier [15]. This is due to the fact that the alteration of mitochondrial function in the neurons promotes mitochondrial respiratory chain complex deficiency, directly affecting neuronal capacity to produce ATP. The lack of energy production adds to an increase in the need for energy, associated with axonal demyelination in patients [16]. This imbalance between the need and contribution of energy leads to higher apoptosis and an increase in the production of reactive oxygen species, as previously indicated [17]. Consequently, thiamine deficiency generates neurological manifestations [18] involving neurodegeneration and symptoms of depression [15]. Likewise, several studies have shown an inverse association between thiamine levels and depression [19] related to increased tyrosine hydroxylase in areas of the brain resulting from oxidative stress [20,21], which is reversed by administering thiamine [22].

Therefore, vitamin B1 is essential for neuronal function, and its deficiency in a person’s diet is related to a greater predisposition to depression [23]. In addition, following a balanced diet that guarantees an adequate supply of this vitamin is postulated as an alternative to improving symptoms of depression [24]. However, patients with this disease face limitations when complying with a balanced food intake as MS symptoms can interfere with adequate nutrition, due in particular to fatigue, cognitive impairment, swallowing problems, mobility restrictions, and high levels of disability. As a result, food preparation and eating becomes compromised, and there is an increase in consuming fast food and high-calorie density foods directly related to an unbalanced diet [25].

Based on the above evidence, the aim of this study is to determine the balance of food intake related to vitamin B1 and its relation to depression in a population with MS.

## 2. Materials and Methods

A cross-sectional, observational descriptive study was conducted.

### 2.1. Subjects

**Figure 1:**
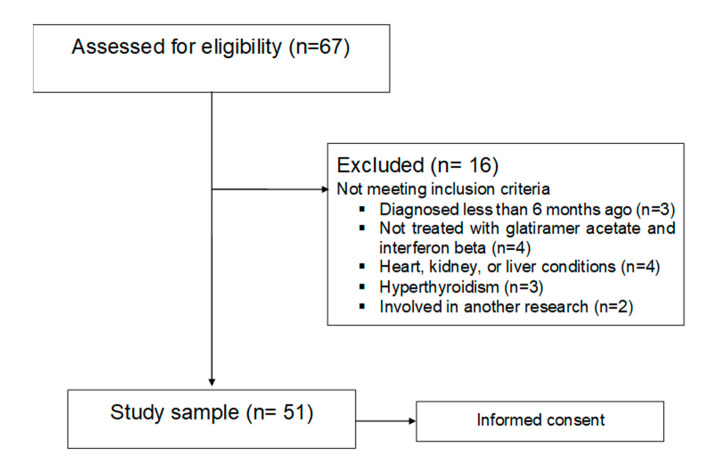
Enrollment flow diagram.

A population sample made up of male and female patients, with an average age of 47 years and with relapsing-remitting or secondary-progressive multiple sclerosis was obtained. The main national MS associations were contacted in the province of Valencia to obtain said sample. Their members were informed accordingly about the nature of the study. The following selection criteria were applied to all 67 people (19 men and 48 women) interested in participating in the study: individuals over 18 years of age who were diagnosed with MS at least six months prior to the start of this study and treated with glatiramer acetate and interferon beta. Exclusion criteria included pregnant or breastfeeding women, patients with tracheostomy, stoma or short bowel syndrome, patients with dementia, as well as those with evidence of alcohol or drug abuse, those with myocardial infarction, heart failure, heart dysrhythmia, symptoms of angina or other heart conditions, cases of patients with kidney conditions with creatinine levels twice as high as normal markers, those with elevated liver markers three times higher than normal or with chronic liver disease, hyperthyroidism, acromegaly, patients with polycystic ovary syndrome, and MS patients who were involved in other research for experimental drugs or treatments. Once the selection criteria were applied, 16 patients were excluded. The remaining patients took part in the study after signing an informed consent form (Figure 1).

### 2.2. Statistical Analysis

A statistical analysis was performed with the SPSS v.23 (IBM Corporation, Armonk, NY, USA) tool. The first step aimed to estimate the distribution of the variables investigated through statistical methods to assess normality, including the Kolmogorov–Smirnov test. This analysis demonstrated the non-normal distribution of all the scale variables studied. Therefore, a two-tailed Spearman′s test was used for the correlation analysis. A *p*-value below 0.05 was considered significant. Data are presented as mean ± standard deviation, or the number of patients and percentage.

### 2.3. Measurements

Measurements used for this study described as follows were carried out individually for each patient by neurologists specializing both in the scales and patients with MS, along with professional nutritionists. All patients had an appointment on the same day at the same time. The neurologists gave them a depression questionnaire at 9 a.m. At 11.30 a.m. the nutritionists carried out a food frequency questionnaire, followed by giving each patient a form to fill in at home regarding their food consumption over seven days. Each participant returned the form by email after the seven days.

The Beck Depression Inventory II (BDI-II) is a questionnaire used to assess symptoms of depression in a patient, mainly of a cognitive type, although physiological, emotional, and motivational types are also considered. Some symptoms included in this version are agitation, feelings of worthlessness, difficulty in concentrating, and loss of energy. The questionnaire contains a symptom of depression for each item and four alternative statements for each, ordered from least to most severe. Each evaluated patient must choose the sentence that best reflects how they felt during the week, including the day they filled out the questionnaire, from 21 questions with four alternative statements. The total score is obtained and ranges from 0 to 63, which quantifies the presence and severity of depression symptoms after assigning each item a score of 0 to 3, depending on the chosen alternative, and after calculating the total score of each item [26].

A description of dietary habits was expected from each patient, and at the beginning of the study, participants completed a Food Frequency Questionnaire (FFQ) [27] to determine the frequency of weekly and monthly food consumption in order to obtain a dietary/nutritional history. Simultaneously, each subject recorded their food consumption over seven days. For that purpose, they were provided with a series of instructions on weight and portion measurements, as well as cooking methods. A nutritional calibration of the macro and micronutrients consumed by each individual was performed with the collected data by using the Easy diet-Programa de gestión de la consulta^®^ software, paying particular attention to food consumed that contained vitamin B1 (whole grains, bread, beef, poultry, liver, fish, nuts, beans, and fortified foods) [28]. The obtained results were used to calculate the average nutritional intake of each nutrient. Additionally, in order to assess whether the diet was adequate, the dietary reference intake (DRI) from “Ingestas de referencia para la población Española” [29] and “Consenso de la Sociedad Española de Nutrición Comunitaria (SENC)” [30] were adopted as guidelines.

The Expanded Disability Status Scale (EDSS) is used to assess functional disability in multiple sclerosis patients [31]. It is an ordinal scale based on a neurological examination of the eight functional systems (pyramidal, cerebellar, brainstem, mental, sensory, visual, bowel, and bladder), together with assessing walking capacity. It provides a disability index ranging between 0 and 10, 0 being considered as normal health and 10 as death by MS.

### 2.4. Ethical Concerns

All patients included in this study signed a consent form after being informed of the procedures and the nature of the study, which was conducted in accordance with the Helsinki Declaration [32], prior to approval of the protocol by the Human Research Committee of the University of Valencia of the Experimental Research Ethics Committee (procedure number H1512345043343).

## 3. Results

After applying the selection criteria specified in the Material and Methods section, and considering the withdrawal of 16 subjects, a sample of 51 MS patients with an average weight of 71.2 kg was analyzed. Their sociodemographic and clinical characteristics are shown in Table 1.

Vitamin B1 deficiency has been associated with the presence of depression. In the study population, after carrying out the necessary calculations as previously indicated, the mean vitamin B1 intake was 1.42 ± 35 mg/day, similar to that published for the same age range in the study by Mielgo-Ayuso et al. in 2018 [33]. However, an eating imbalance could also promote the onset of emotional disturbances, such as depression, related to the poorer quality of life and functional worsening typically observed in MS patients [34,35]. Accordingly, loss of function in depressed patients is closely related to unbalanced diets characterized mainly by high amounts of simple carbohydrates, leading to a further worsening of depression [36]. The study population presents, on the one hand, a functional deficit characteristic of the disease (Table 1) and, on the other hand, a dietary imbalance as already reported in a previous paper [37]. This imbalance is outlined due to a lower than recommended intake of total carbohydrates, which could interfere with the activity of vitamin B1 as its function is to metabolize carbohydrates to obtain glucose as a source of neuronal energy [14]. Furthermore, most carbohydrates consumed are simple carbohydrates, among which those from ultra-processed foods predominate. These products provide poor-quality simple carbohydrates and, particularly, from greater to lesser consumption in our MS population, included dairy desserts, juice, snacks, pastries, chocolate bars, soft drinks and fermented alcohol (Table 2). The intake of these types of products, which are characterized by a high energy input and high caloric density, have already been associated with patients with MS [25] and, therefore, depression [38,39].

**Figure 2:**
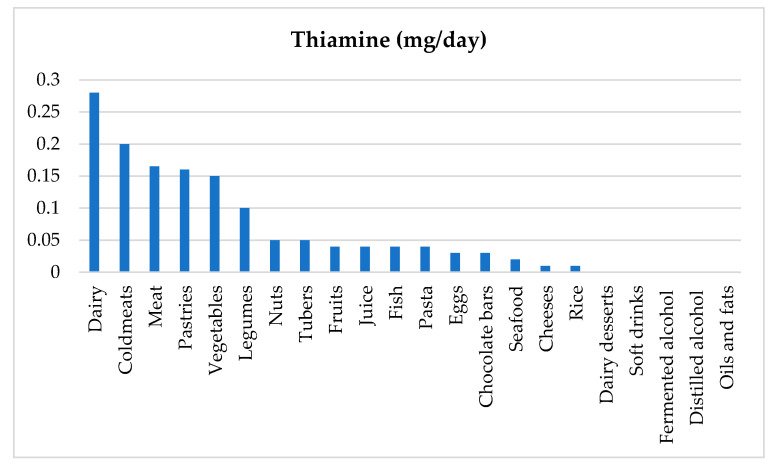
Nutritional sources of thiamine in the study population.

After analyzing the nutritional sources that provided thiamine to our population (Figure 2), these were to a large extent equally ultra-processed products such as cold meats or pastries. However, the main sources (after meat and meat products) consumed by healthy people of the same age are cereals such as pasta or rice, which are rich in complex carbohydrates [33].

Therefore, there should be a balance between the amount and quality of carbohydrates consumed, as well as the intake and sources of thiamine. Otherwise, neuronal function could be impaired by emotional disturbances. This could be a possible reason to justify, on the one hand, the negative correlation observed between total carbohydrate intake and the presence of depression (Table 3), and, on the other hand, the negative correlation between thiamine intake calculated from the nutritional sources of our study population and the levels of depression (Table 3).

**Figure 3:**
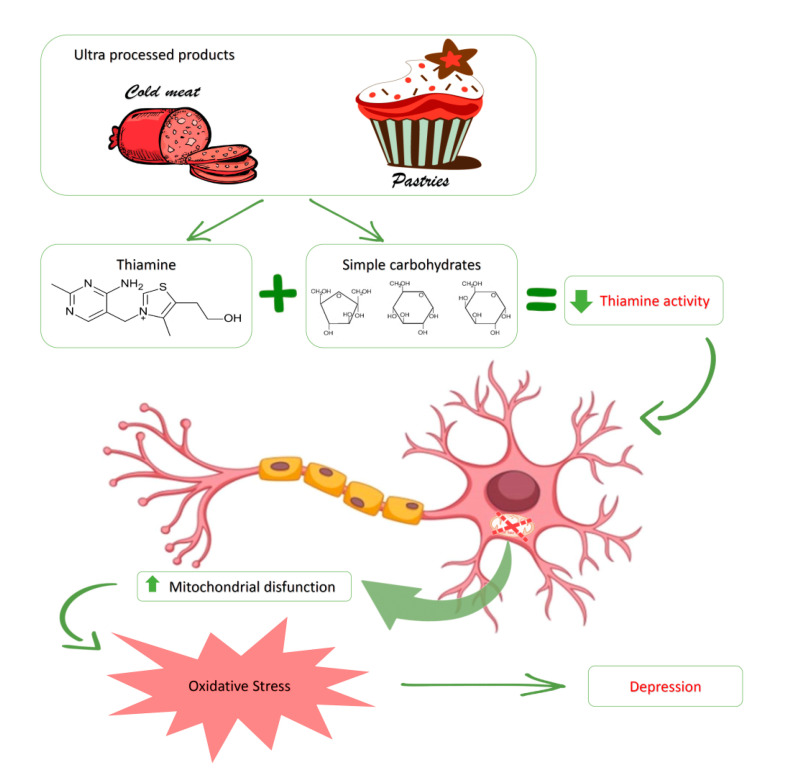
Activity of vitamin B1 (thiamine) and its role in the presence of depression in MS patients, related to the intake of ultra-processed food. A high consumption of ultra-processed foods, among which we can highlight cold meat and pastries, is the main source of thiamine and large quantities of simple carbohydrates that patients with multiple sclerosis ingest (within a diet low in total carbohydrates). As a result, metabolic function of vitamin B1 is altered, therefore reducing its activity. This gives rise to an increase in mitochondrial dysfunction on a neuronal level, which favors an increase in oxidative stress directly associated with depression that is characteristic of this disease.

After debating the results, we can state that depression related to neuronal damage consisting of myelin deterioration may indicate that these types of patients, despite ingesting adequate levels of thiamine, need balanced diets with sources of vitamin B1 similar to those described for a healthy Spanish population [33] or with intakes higher than those recommended, given that thiamine supplementation is non-toxic. [40]. In this sense and based on the possible processes shown in Figure 3 after debating these results, an increase in thiamine consumption within the diet would improve activity based on the correct metabolism of carbohydrates. As a result, this could cause a deceleration of neuronal loss, alleviating depression related to the reduction of neurogenesis of the damaged hippocampus and [22] associated with depression in MS patients [41].

## 4. Discussion

These results indicate the importance of determining the intake of vitamin B1 and its main nutritional sources in patients with MS with severe cases of depression. In this sense, administering balanced diets low in ultra-processed foods, rich in thiamine from adequate nutrients, and with sufficient amounts of total carbohydrates could be a therapeutic alternative considered in order to improve the emotional state in these patients. Despite these results, our study has some limitations that should be considered. The main limitation is the difficulty in specifying the consumption of foods with thiamine and, consequently, the daily intake of vitamins by the patients. In addition, it would be necessary to replicate the study with a larger population of patients to determine possible correlations between thiamine intake and stress biomarkers such as the hormone cortisol. This would also allow consideration into the possibility of administering a balanced diet with high levels of thiamine in order to determine and evaluate any changes in the levels of depression.

## 5. Conclusions

Nutritional intake related to thiamine activity in an MS population shows imbalances characterized by a below-recommended total carbohydrate intake that is composed mainly of simple carbohydrates. In addition, both simple carbohydrates and thiamine derive primarily from the consumption of ultra-processed products. This evidence suggests that patients who consume fewer total carbohydrates and thiamine are those who show higher levels of depression which is characteristic of the disease.

## Figures and Tables

**Table 1:** Socio-demographic and clinical characteristics of the population of the study.

		Count	%
MSType	Relapsing-Remitting	37	72.5%
	Secondary-Progressive	14	27.5%
Gender	Man	15	29.4%
	Woman	36	70.6%
		Mean	SD
Age (years)		47.04	12.00
Time since diagnosis (years)		12.98	9.12
EDSS		3.56	2.00

MS: Multiple sclerosis; EDSS: Expanded Disability Status Scale; SD: standard deviation.

**Table 2:** Dietary habits of the study population related to the consumption of nutrients rich in simple carbohydrates.

Main Nutrients with Simple Carbohydrates	Number of Monthly Intakes	
	Mean	SD
Dairy	16.81	12.89
Cheeses	11.37	9.86
Dairy desserts	0.78	2.49
Vegetables	19.26	8.81
Fruit	22.67	8.52
Juice	9.93	12.02
Snacks	2.89	3.89
Pastries	12.41	11.19
Chocolate bars	10.19	10.50
Soft drinks	5.81	8.87
Fermented alcohol	6.15	7.39
Distilled alcohol	0.15	0.46

SD: Standard deviation.

**Table 3:** Correlations of depression with vitamin B1 and total carbohydrate intake in the study population.

Variable	VitaminB1		Total Carbohydrate Intake	
	Coef.	Sig.	Coef.	Sig.
Depression (BDI-II)	−0.377	0.031 *	−0.339	0.043 *

BDI-II: Beck Depression Inventory II; Coef.: Spearman Correlation Coefficient; Sig.: Signification; *: statistically significant differences *p*< 0.05.
